# Elements of Surgery

**Published:** 1831-01

**Authors:** 


### BIBLIOGRAPHICAL NOTICE.

*Elements of Surgery.* By ROBERT LISTON, Fellow of the Royal College of Surgeons in London and Edinburgh, &c. Part I.—8vo. pp. 318. London: Longman and Co. 1831.

In this work, the author proposes to reduce the heads of his Surgical Lectures into a compendium, or guide, for those students who repair to Edinburgh for instruction. To them it will, therefore, be especially useful; but all surgical students will study it with advantage. Mr. Liston first discusses the general principles which ought to guide the practitioner in the management of constitutional disturbance, however occasioned. The observations introduced to illustrate the doctrines inculcated, are given as briefly as is consistent with an accurate detail of symptoms and results. The descriptions of particular diseases have been sketched and finished from nature. The phenomena of morbid action are clearly and simply detailed, as well as the various changes which it produces. Perspicuous rules are given for the treatment of surgical diseases, and for performing the operations required for their alleviation or cure. When this work is completed, it will form a most useful initiatory study in the science of surgery, and will effectually prepare the learner to enter upon the perusal of other works of a more comprehensive character. Speculative doctrines are judiciously avoided: an elementary work should be confined to facts.

